# Mindestmengen in der chirurgischen Behandlung des Lungenkarzinoms: Ein Meinungsbild von in Deutschland aktiven Thoraxchirurgen zur Einführung einer Mindestmengenregelung für die chirurgische Therapie des Lungenkarzinoms

**DOI:** 10.1007/s00104-020-01185-9

**Published:** 2020-05-07

**Authors:** Tobias Robold, Michael Ried, Reiner Neu, Hans-Stefan Hofmann

**Affiliations:** Abteilung für Thoraxchirurgie, Universitätsklinikum Regensburg, Franz-Josef-Strauß Allee 11, 93053 Regensburg, Deutschland

**Keywords:** Qualitätssicherung, Thoraxchirurgie, Lungenkarzinom, Versorgungsqualität, Krankenhaussterblichkeit, Quality assurance, Thoracic surgery, Lung cancer, Quality of care, In-hospital mortality

## Abstract

**Hintergrund:**

Im Rahmen der aktuellen Diskussion des G‑BA zur Einführung neuer Mindestmengenregelungen (MMR) in Deutschland untersucht die vorliegende Studie das Meinungsbild aktiver Thoraxchirurgen zu Mindestmengen (MM) bei der operativen Behandlung des Lungenkarzinoms.

**Methoden:**

Die Auswahl der thoraxchirurgischen Zentren für die Onlinebefragung erfolgte auf Basis des Krankenhausverzeichnisses 2017 (Bundesamt für Statistik), der Lungenkrebszentren (Deutsche Krebsgesellschaft), der zertifizierten Kompetenzzentren Thoraxchirurgie (Deutsche Gesellschaft für Thoraxchirurgie), der Kliniken mit thoraxchirurgischem Schwerpunkt und der deutschen Universitätskliniken. Abgefragt wurde der potenzielle Einfluss einer MMR auf die Ergebnisqualität, Versorgungsqualität, ökonomische Aspekte und auf die Versorgungsstruktur. Des Weiteren wurde eine Empfehlung für eine MM gefordert und aktuelle Ausnahmeregelungen bewertet.

**Ergebnisse:**

Es wurden 145 Kliniken (Rücklaufquote 85 %) mit 454 Thoraxchirurgen (Rücklaufquote 54 %) kontaktiert. Bei hoher Akzeptanz von MM zur Verbesserung der Ergebnisqualität erwarten 78,4 % der befragten Operateure eine Zentralisierung der chirurgischen Versorgung, welche jedoch nach Aussage von 70,1  % zu keiner Verschlechterung der Versorgung von Lungenkrebspatienten führen würde. Etwa 46,1 % der Teilnehmer rechnen mit einer ökonomischeren Versorgung und 83,3 % sprachen sich für die Einführung einer MMR mit einer durchschnittlichen MM von 67 anatomischen Lungenresektionen pro Jahr und pro Zentrum aus.

**Schlussfolgerung:**

Eine MMR zur chirurgischen Therapie des Lungenkarzinoms findet unter aktiven Thoraxchirurgen eine hohe Akzeptanz. Die geforderte MM (*n* = 67) liegt etwas unter der Vorgabe für chirurgische Primärfälle eines zertifizierten Lungenkrebszentrums.

## Abstract (en)

**Background:**

The Federal Joint Committee (G‑BA) is currently discussing the introduction of new minimum volume regulations (MVR) in Germany. The present study examined the current opinions of active thoracic surgeons regarding minimum volumes (MV) for the surgical treatment of lung cancer.

**Methods:**

The participating centers for the online survey were identified on the basis of the thoracic surgery departments in the 2017 hospital directory (Federal Statistical Office), lung cancer centers (German Cancer Society), certified centers of excellence for thoracic surgery (German Society for Thoracic Surgery), hospitals with a focus on lung surgery and German university hospitals. They were asked about the potential effects of MVR on the quality of results and quality of care, economic aspects and the structure of care. Furthermore, a recommendation for MV was requested and possible provisions for exemption were evaluated.

**Results:**

A total of 145 hospitals (response rate 85%) with 454 thoracic surgeons (response rate 54%) were surveyed. The results showed a high degree of approval for MV to improve the quality of results and 78.4% of the surgeons surveyed expected it to result in centralization of surgical care, although this would not lead to a deterioration in care according to 70.1% of the participants. Approximately 46.1% of the participants expected care to become more economical and 83.3% supported the introduction of an MVR, with the average recommended MV being 67 anatomical lung resections per center per year.

**Conclusion:**

An MVR for the surgical treatment of lung cancer met with a high degree of approval among active thoracic surgeons. The MV that was called for (*n* = 67) was slightly below the prerequisite for primary surgical cases at a certified lung cancer center.

Der Gemeinsame Bundesausschuss (G-BA) prüft derzeit die Einführung einer Mindestmengenregelung (MMR) für Lungenkrebsoperationen in Deutschland, zur potenziellen Verbesserung der Qualität des Behandlungsergebnisses. Die vorliegende Studie untersuchte dabei das aktuelle Meinungsbild aktiver Thoraxchirurgen zu Mindestmengen (MM) bei der operativen Behandlung des Lungenkarzinoms mit Empfehlung einer konkreten MM und Bewertung der aktuell gültigen Ausnahmeregelungen.

## Hintergrund

In der Diskussion um die Zukunftsfähigkeit der Krankenhausversorgung werden zum notwendigen Strukturwandel diverse Ziele diskutiert [1]. Mindestmengen (MM) spielen dabei zur Förderung von Konzentrationsprozessen inklusive Erhöhung der Prozess- und Ergebnisqualität eine wesentliche Rolle. Mit dem Beschluss vom 19.07.2018 hat der Gemeinsame Bundesausschuss (G-BA) die Einleitung eines Beratungsverfahrens zur Einführung von Mindestmengen für die chirurgische Behandlung des Lungenkarzinoms veranlasst. Das Lungenkarzinom ist in Deutschland die Tumorentität mit der höchsten Letalität von 45.804 Sterbefällen und einer Inzidenz von 57.460 Neuerkrankungen pro Jahr (Daten aus dem Jahr 2016; [2]). Ein Zusammenhang zwischen dem chirurgischen Behandlungsvolumen und der Versorgungsqualität beim Lungenkarzinom wurde bereits in nationalen und internationalen Publikationen nachgewiesen [3–5].

Die anatomische Lungenresektion (Segmentresektion, [Bi-]Lobektomien und Pneumonektomie) gilt als Standard bei der onkologisch-gerechten operativen Versorgung eines Lungenkarzinoms [6], daher wurde auch die Anzahl dieser Eingriffe als geeigneter Qualitätsparameter definiert.

Die Aufarbeitung von Daten aus Deutschland auf Basis der DRG(„diagnosis related group“)-Statistik der Jahre 2005 bis 2015 zeigte nahezu eine Verdopplung der Krankenhausletalität in Zentren mit einem Behandlungsvolumen von weniger als 25 anatomischen Lungenresektionen pro Jahr (5,7 %) bei der Hauptdiagnose Lungenkarzinom im Vergleich zu Zentren mit einem Volumen von mehr als 75 anatomischen Lungenresektionen pro Jahr (3,0 %; [7]).

Die chirurgische Qualität objektiv zu beurteilen, ist jedoch schwierig und lässt sich nicht allein auf die Arbeit im Operationssaal reduzieren. Neben der unmittelbaren Ergebnisqualität müssen daher auch die Dimensionen der Strukturqualität und der gegebenen Prozessabläufe in der Bewertung berücksichtigt werden [8].

Die Auswahl von Qualitätsindikatoren (QI) mit Festlegung von Reverenz- bzw. Grenzwerten ist komplex und kann nur dann einen Beitrag zur Qualitätssicherung liefern, wenn sie auch von den behandelnden Chirurgen anerkannt und umgesetzt werden [9]. Aus diesem Grund führten wir eine Umfrage unter aktiven Thoraxchirurgen in Deutschland zur Erstellung eines Meinungsbildes zu MM in der Thoraxchirurgie bei der chirurgischen Behandlung des Lungenkarzinoms durch.

## Methoden

Die Auswahl der kontaktierten Zentren erfolgte anhand des Krankenhausverzeichnisses 2017 (Bundesamt für Statistik), der assoziierten thoraxchirurgischen Zentren der Lungenkrebszentren nach der Deutschen Krebsgesellschaft (DKG), den Kompetenzzentren für Thoraxchirurgie der Deutschen Gesellschaft für Thoraxchirurgie (DGT) sowie den Schwerpunktzentren für Thoraxchirurgie (verfügbar über den Onlineauftritt der DGT; Stand 01/2019), den thoraxchirurgischen Abteilungen der deutschen Universitätskliniken und durch eine zusätzliche manuelle Recherche [10]. Es wurden die online verfügbaren Kontaktdaten der Mitarbeiter des jeweiligen Zentrums zur Kontaktaufnahme genutzt, sofern sie erkennbar einer thoraxchirurgischen Tätigkeit zugeordnet werden konnten. Eine Einladung zur Teilnahme an der Umfrage wurde an 454 Chirurgen in 145 Zentren verschickt. Einmalig wurde elektronisch an die Umfrage erinnert.

Der Befragungszeitraum erstreckte sich vom 17.01.2019 bis 26.02.2019. Der Fragenkatalog umfasste insgesamt 15 Fragen. Im ersten Teil wurde nach dem Ausbildungsstand sowie nach Struktur und Versorgungszahlen am Zentrum des Teilnehmers gefragt. Der zweite Teil beschäftigte sich mit allgemeinen Aspekten zum Thema MM, während abschließend neben thoraxchirurgisch spezifischen Aspekten auch um eine Empfehlung für Grenzwerte von MM für anatomische Lungenresektionen beim Lungenkarzinom gebeten wurde. Die Umfrage wurde als Onlinesurvey zur Verfügung gestellt und über die Plattform SoSci Survey (SoSci Survey GmbH, München, Deutschland) realisiert. Für akademische Umfragen ist die Nutzung des Service kostenfrei. Die verschlüsselte Datenübermittlung und -speicherung mit Serverstandort in Deutschland folgte den Vorgaben der Datenschutz-Grundverordnung (DSGVO) in ihrer aktuellen Fassung.

Die statistische Analyse erfolgte deskriptiv. Häufigkeit und Verteilung der Antworten wurden absolut und prozentual ausgewertet. Zur Analyse von Gruppenvergleichen wurde der Fischer-Exakt-Test/χ^2^-Test genutzt. Verwendet wurde die Statistiksoftware SPSS (IBM SPSS Statistics for Windows, Version 24.0. IBM Corp., Armonk, NY, USA). Das Signifikanzniveau wurde auf *p* = 0,05 definiert.

## Ergebnisse

### Studienteilnehmer

Insgesamt 244 von 454 adressierten Teilnehmern aus 122 Zentren nahmen an der Umfrage teil. Dies entspricht einer Rücklaufquote von 53,7 % der Teilnehmer und 84,7 % der Zentren. Für die Analyse gültige Interviews (>90 % der Fragen beantwortet) leisteten 223 der 244 (94,17 %) Teilnehmer. Etwa 96,5 % der Teilnehmer waren Fachärzte für Thoraxchirurgie (*n* = 193) oder befanden sich in der Fachweiterbildung (*n* = 21). Der Großteil der Teilnehmer stammte aus Zentren mit 6 bis 8 (*n* = 144) bzw. 3 bis 5 (*n* = 40) Fachärzten sowie 1 bis 2 (*n* = 123) oder 3 bis 5 (*n* = 55) Weiterbildungsassistenten für Thoraxchirurgie. Etwa 56 % der Teilnehmer stammten aus Lungenkrebszentren der DKG, 19,7 % aus Zentren mit geplanter Zertifizierung zum Lungenkrebszentrum (sog. Transitstatus), 20,2 % der Teilnehmer waren aus Kompetenzzentren für Thoraxchirurgie (DGT) sowie 26 % aus Organkrebszentren (DKG) ohne Lungenkrebszentrumsstatus und/oder onkologischen Zentren der DKG (37,7 %). Keine Zertifizierung am Standort gaben 9 % der Teilnehmer an.

### Beurteilung der Mindestmenge

**Figure 1:**
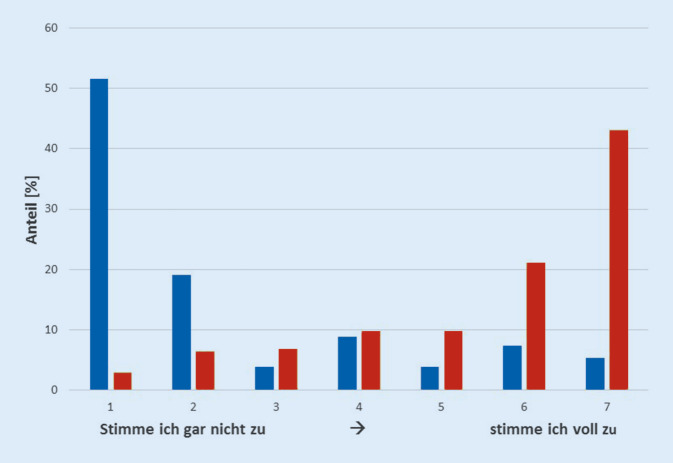

Die Mehrzahl der Teilnehmer halten MM für ein geeignetes Werkzeug zur Verbesserung der Ergebnisqualität, dies gilt sowohl allgemein für MM in der Chirurgie (Zustimmung 69,9 %) als auch für den Fachbereich Thoraxchirurgie im Speziellen (74 %; s. Abb. 1). Zur MMR für planbare stationäre Leistungen wurden durch den G‑BA Ausnahmen (Ausnahmetatbestände (AT)) formuliert, welche auch beim Unterschreiten von MM eine Durchführung der Operation zulassen. Bei neuen MM oder Erhöhung einer bereits geltenden MM gilt eine Übergangsfrist von in der Regel 12 Monaten (maximal 24 Monate). Diese AT wurden überwiegend neutral (29,6 %) oder positiv (43,5 %) bewertet. Ebenso positiv (50,2 %) oder neutral (22 %) wurde die Ausnahmeregelung für den Aufbau neuer Leistungsbereiche mit einem Übergangszeitraum von 24 Monaten und der Reduktion der MM um 50 % im ersten Jahr beurteilt. Negativ beeinflussend (41,7 %) bzw. neutral (24,2 %) wurde der AT zur Notwendigkeit einer flächendeckenden Versorgung gesehen. Die Möglichkeit auch Leistungen außerhalb der planbaren Erbringung (Notfälle) abrechnen zu können, wurde heterogen (negativ 27,8 %, neutral 30,9 %, positiv 36,3 %) beurteilt.

**Figure 2:**
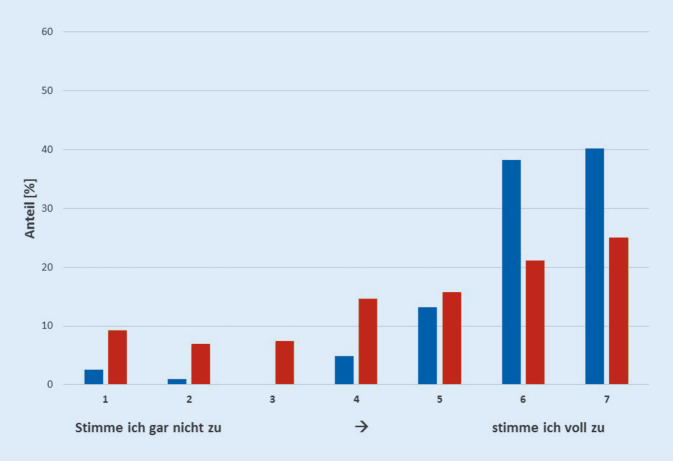

Insgesamt 70,1 % der Teilnehmer stimmten der Aussage nicht bzw. gar nicht zu, dass MM in der Thoraxchirurgie zu einer schlechteren Versorgung von Patienten mit Lungenkarzinom in Deutschland führen. Knapp die Hälfte (46,1 %) der Teilnehmer sahen das Potenzial für eine ökonomischere Versorgung und 78,4 % erwarteten eine Zentralisierung der chirurgischen Versorgung von Patienten mit Lungenkarzinom durch Einführung einer MMR (Abb. 2).

**Figure 3:**
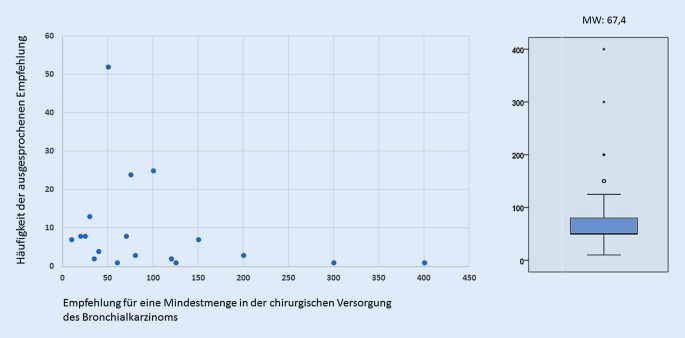

**Figure 4:**
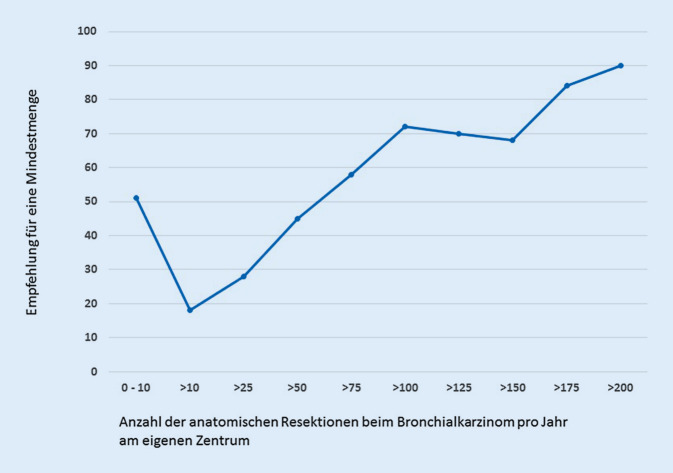

Die große Mehrheit der befragten Thoraxchirurgen (*n* = 170; 83,3 %) sprachen sich für die Einführung von MM in der chirurgischen Behandlung des Lungenkarzinoms aus. Nur 16,7 % der Teilnehmer hielten dies dagegen für nicht sinnvoll. Die Befürworter der Einführung einer MMR gaben im Mittel 67 anatomische Lungenresektionen pro Jahr und pro Zentrum an (Abb. 3). Es zeigte sich ein signifikanter Zusammenhang zwischen der ausgesprochenen Empfehlung und der Anzahl der chirurgischen Primärfälle (anatomische Lungenresektion bei Lungenkarzinom) am eigenen Zentrum (Abb. 4).

## Diskussion

Zahlreiche Publikationen verweisen auf einen Zusammenhang zwischen Leistungsmenge und Qualität des Behandlungsergebnisses bei der chirurgischen Behandlung des Lungenkarzinoms [3–5, 7, 11–13]. Mehr als 80 % der Thoraxchirurgen sprachen sich in der vorliegenden Studie für die Einführung einer solchen MMR in der chirurgischen Behandlung des Lungenkarzinoms aus. Mit 145 befragten thoraxchirurgischen Zentren ist anhand aktueller Aufarbeitungen der DRG-Statistiken davon auszugehen, dass in den befragten Zentren mehr als 80 % der kurativ-chirurgischen Behandlungen bei Patienten mit Lungenkarzinom durchgeführt werden [7]. Die überdurchschnittlich hohe Rücklaufquote (Teilnehmer 53,7 %, Zentren 84,7 %) unterstreicht das hohe Interesse der Thoraxchirurgen an der aktuellen Diskussion zum Thema MM bei der chirurgischen Therapie des Lungenkarzinoms. Die Akzeptanz von MM durch die betroffenen Chirurgen ist besonders wichtig, da beispielsweise im Jahr 2017 39,7 % der Kliniken, die heute schon von einer MMR (z. B. Kniegelenksoperationen, Ösophagusresektionen, Leber- und Nierentransplantation) betroffenen sind, die gesetzlichen Mindestfallzahlen nicht erreichten [14].

Die Mehrheit der Thoraxchirurgen sieht durch die Einführung von MM in der chirurgischen Versorgung von Lungenkarzinompatienten keine Gefahr einer Verschlechterung der Behandlungssituation in Deutschland. Es wird jedoch eine Zentralisierung der Versorgung (78,1 %) mit dem Potenzial einer ökonomischeren chirurgischen Behandlung (46,1 %) von Patienten mit Lungenkarzinom erwartet. Die Empfehlung der Thoraxchirurgen hinsichtlich der Höhe der MM lag bei 67 anatomischen Lungenresektionen pro Jahr. Diese Empfehlung liegt im Vergleich zu den derzeit geltenden MM für andere operative Eingriffe sehr hoch, aber immer noch unterhalb der geforderten MM anatomischer Lungenresektionen pro Jahr zur Zertifizierung zum Lungenkrebszentrum (DKG), die bei ≥75 chirurgischen Primärfällen liegt [15]. Seit dem Jahr 2016 werden hier jedoch Resektionen bei allen bösartigen Tumorentitäten in der Lunge (z. B. auch Lungenmetastasen) zur operativen Expertise gezählt (ICD-10 C‑Diagnose inklusive ICD-10 C34). Bezogen auf das Abrechnungsjahr 2015 wären von o. g. Empfehlung mindestens 246 Abteilungen/Kliniken mit ≤50 durchgeführten anatomischen Lungenresektionen bei der Diagnose Lungenkarzinom mit einem Volumen von 3432 Operationen betroffen. Bei den sog. Low-volume-Kliniken (≤25 anatomische Lungenresektionen pro Jahr) verteilten sich im gleichen Jahr 1576 Eingriffe auf 194 Abteilungen [7, 16]. Die Verteilung Low- vs. High-volume-Kliniken ist dabei bundesweit sehr unterschiedlich. So werden nach Angaben des Wissenschaftlichen Instituts der AOK heute schon in Berlin mehr als 97 % der Lungenkrebspatienten in Kliniken mit mehr als 75 Eingriffen pro Jahr operiert, in Mecklenburg-Vorpommern sind es hingegen nur 25 %.

Betrachtet man die derzeit bestehenden MM für operative Verfahren, so ist die Einführung einer MM, welche sich an den DKG-Kriterien für Lungenkrebszentren orientiert (*n* ≥ 75) eher unwahrscheinlich. Aus gesundheitspolitischen Aspekten stellt sich die Frage, ob eine MM in der Höhe von 25 Eingriffen nicht ausreicht, um eine „Gelegenheitschirurgie“ auszuschließen. Aktuell werden ca. 14 % der anatomischen Resektionen beim Lungenkarzinom durch Kliniken mit <25 anatomischen Lungenresektionen pro Jahr durchgeführt [7, 16]. Diese Einheiten bieten thoraxchirurgische Leistungen in der Regel ergänzend zu einem meist allgemein-/viszeralchirurgischen oder herzchirurgischen Leistungsschwerpunkt der Klinik an.

Die European Surgical Association (ESA) betont in einem Konsensuspapier neben dem Krankenhausfallaufkommen auch die Bedeutung des Operationsvolumens des Chirurgen und seine Spezialisierung [17]. Fachlichen Standards und ein Mindestbehandlungsvolumen sind dabei ein Annäherungsmaßstab für bestimmte Charakteristika der Prozess- und Strukturqualität. Bei der Beurteilung von Qualität muss speziell auch die Versagerrate nach dem Auftreten einer Komplikation („failure-to-rescue“) bewertet werden. Auch bei gleicher Komplikationsrate bieten sich für Zentren mit hohem Eingriffsvolumen potenzielle Vorteile durch die Erfahrung des Personals vor Ort, die Verfügbarkeit von Spezialisten rund um die Uhr oder eine spezialisierte Infrastruktur [18].

Nur wenige Studien fragen nach der Beziehung zwischen Fallaufkommen des Chirurgen und Ergebnissen nach Lungenresektion. Aktuelle Untersuchungen zeigen zwar einen wesentlichen Einfluss chirurgischer Fallzahlen auf die Krankenhaus- bzw. 30-Tage-Letalität [19, 20] und konnten auch einen positiven Einfluss auf das Pneumektomierisiko nachweisen [21], eine evidenzbasierte Aussage zu potenziellen Schwellenwerten lässt sich aber derzeit nicht ableiten.

Die Spezialisierung des Chirurgen wirkt sich ebenfalls positiv auf die Ergebnisse der Lungenresektion aus [22, 23]. Die Datenlage ist aber ebenfalls nicht ausreichend, um Grenzwerte zu formulieren, insbesondere da die Literatur nicht die Ausbildung und den Spezialisierungsgrad in Deutschland abbildet.

International wird von der Leapfrog-Gruppe in den USA für 11 Operationen sowohl eine Mindestmenge für die Krankenhäuser als auch für die Operateure gefordert. Für Lungenresektionen bei Karzinom beträgt das Krankenhausvolumen ≥40 Eingriffen pro Jahr bei einem Chirurgenvolumen von ≥15 Eingriffen pro Jahr [8]. Weder in den aktuellen gesetzlichen MMR in Deutschland, noch in der Diskussion zur Einführung von MMR in der Versorgung des Lungenkarzinoms sind individuelle Vorgaben vorgesehen. Lediglich in die Strukturqualität der Lungenkrebszentren ist der Umfang an Mindestoperationen für den Thoraxchirurgen erfasst.

Es sollte ebenfalls diskutiert werden, ob die alleinige Anzahl an Operationen für die Beurteilung der Versorgungsqualität ausreichend ist. Neben der Ergebnisqualität sollten auch die Aspekte der Struktur- und Prozessqualität berücksichtigt werden. Um Qualität messen zu können, muss zunächst der Anspruch an gute Qualität festgelegt werden, insbesondere da durch den G‑BA eine neue Ausnahmeregelung für „hohe Qualität“ eingeführt wurde, die eine Genehmigung zur Durchführung auch unterhalb der MMR zulässt. Allerdings müssten dafür messbare Qualitätsindikatoren (QI) zur Bewertung herangezogen werden können, welche als Surrogat-Parameter für in der Regel Teilbereiche der gewünschten Qualität stehen. Einzelne QI können naturgemäß in der Regel nur Teilaspekte des Qualitätsanspruches abbilden. Somit wird die Verwendung mehrerer QI im Sinne von Qualitätsindikatorprofilen empfohlen [24]. Bewertungssysteme zur Beurteilung guter Qualität sind in der aktuellen MMR für keine der bereits gültigen MM verankert. Die Anforderungsprofile zur Zertifizierung zum Lungenkrebszentrum der DKG oder zum Kompetenzzentrum für Thoraxchirurgie der DGT stellen derzeit die verbreitetsten QI-Profile zur QS im Fachbereich Thoraxchirurgie in Deutschland dar.

Mit der Einführung von MMR für Krankenhäuser in Deutschland im Jahr 2004 wurden auch Ausnahmetatbestände (AT) formuliert. Diese ermöglichen die Abrechnung von MM-Operationen auch unterhalb der MM-Vorgaben. Die AT sind für alle MMR gültig und würden somit auch eine potenzielle Regelung für anatomische Resektionen beim Lungenkarzinom betreffen. Die aktuellen AT wurden von den Umfrageteilnehmern der Studie überwiegend positiv bewertet. Dies betrifft zum einen die AT für „erstmalige Erbringung einer Leistung (…) oder die erneute Erbringung nach mindestens 24-monatiger Unterbrechung“ [25]. Auch die aktuelle Übergangsfrist von in der Regel 12 Monaten (max. 24 Monaten) bei Neueinführung einer MM bzw. Erhöhung wurde durch die befragten Thoraxchirurgen überwiegend neutral bzw. positiv bewertet. Eine weitere Ausnahme betrifft die Abrechenbarkeit von Notfällen, welche in unserer Studie überwiegend nur neutral bewertet wurde. Da es sich bei der chirurgischen Versorgung des Lungenkarzinoms in den meisten Fällen um planbare Operationen handelt, spielt diese Ausnahme quantitativ eine eher untergeordnete Rolle. Für die Umsetzung des AT zur Sicherstellung einer flächendeckenden Versorgung der Bevölkerung bedurfte es bis 2017 zur Verifizierung des Bedarfs eine Genehmigung seitens der zuständigen Landesbehörde. Diese in unserer Umfrage überwiegend negativ bewertete Ausnahme findet sich auch seit dem Jahr 2018 nicht mehr in der aktuellen MMR [25]. Dennoch kann die Landesbehörde weiterhin Ausnahmegenehmigungen zur Aussetzung der MMR für einzelne Institutionen erteilen.

Für die Umgehung bestehender MMR konnten neben den schon diskutierten AT auch bestandene Abrechnungsmodalitäten genannt werden. Durch die Novellierung der MMR durch den G‑BA zum 01.01.2018 muss der Krankenhausträger nun vor Erbringung der Leistung gegenüber der Krankenkasse darlegen, dass die vorgegebenen MM im folgenden Kalenderjahr erreicht werden können. Ist dies nicht möglich, ergibt sich kein Vergütungsanspruch durch die Krankenkassen [25–27].

Am 16.08.2018 wurde das Institut für Qualität und Wirtschaftlichkeit im Gesundheitswesen (IQWiG) durch den G‑BA mit einer systematischen Literaturrecherche inklusive Evidenzbewertung zum Zusammenhang der Leistungsmenge und der Qualität des Behandlungsergebnisses bei der chirurgischen Behandlung des Lungenkarzinoms beauftragt. Die Ergebnisse der Literaturrecherche des IQWiG (Stand 08.10.2019) wurden am 06.11.2019 veröffentlicht. Es wurden 23 Beobachtungsstudien in die Analyse eingeschlossen, wobei nur 19 Studien verwertbare Daten für die Darstellung und Bewertung des Zusammenhangs zwischen der Leistungsmenge und der Qualität des Behandlungsergebnisses bei der chirurgischen Behandlung des Lungenkarzinoms beinhalteten [28]. Acht der 23 Studien fanden sich bereits in der Metaanalyse von 2012, wobei keine der Arbeiten Aussagen zur Auswirkung konkret in die Versorgung eingeführter Mindestfallzahlen auf die Qualität der Behandlungsergebnisse ermöglichten [13]. Durch den überwiegend positiv nachgewiesenen Zusammenhang zwischen der Leistungsmenge und der Qualität des Behandlungsergebnisses im Gesamtüberleben, therapieassoziierter Letalität und Versterben im Krankenhaus wird von einer höheren Sterblichkeit bei geringerer Leistungsmenge ausgegangen. Den herangezogenen Studien wird jedoch nur eine niedrige Aussagekraft der Ergebnisse attestiert. Zu den wichtigen Zielgrößen wie 30-/90-Tage-Letalität und therapieassoziierte Letalität konnten u. a. aufgrund unscharfer Definitionen der Zielgrößen in den einzelnen Studien keine konsistenten Aussagen getroffen werden. Für weiter Zielgrößen wie krankheitsfreies Überleben, Infektionen, therapiebedingte Komplikationen oder gesundheitsbezogene Lebensqualität konnten bei fehlenden Daten keine Auswertungen erfolgen [28].

## Resümee

Durch die Beauftragung des IQWiG durch den G‑BA zur systematischen Literaturrecherche zum Zusammenhang zwischen Leistungsmenge und Qualität des Behandlungsergebnisses wurde ein wesentlicher Schritt zur Diskussion über die Einführung einer MMR zur chirurgischen Versorgung des Lungenkarzinoms (Thoraxchirurgie bei Lungenkarzinom) in Deutschland gemacht. Analog zu bereits bestehenden Publikationen wird in der abschließenden Beurteilung des IQWIG „von einer höheren Sterblichkeit bei geringerer Leistungsmenge ausgegangen“. Einschränkend wird auf die insgesamt geringe Datenlage/-qualität verwiesen, welche keine Aussagen zu konkret in die Versorgung eingeführte Mindestfallzahlen zulassen. Diese aktuelle Umfrage unter Thoraxchirurgen (244 Teilnehmer aus 122 Zentren in Deutschland) zeigt, dass unter den aktiv in die Versorgungssituation des Lungenkarzinoms integrierten Thoraxchirurgen eine hohe Zustimmung (83,3 %) zur Einführung einer MMR mit den aktuell gültigen Ausnahmeregelungen besteht. Es wurde von diesen eine durchschnittlich MM von 67 anatomischen Lungenresektionen pro Jahr und Zentrum empfohlen. Für die Beurteilung der Qualität einer Versorgungseinrichtung sollte jedoch die Verwendung eines einzelnen Qualitätsindikators nicht ausreichend sein. Hier bieten Indikatorprofile aus Zertifizierungsprozessen (Definition einer Struktur- und Prozessqualität) eine zusätzliche, validere Grundlage. Als Kennzahl bietet die Anzahl der durchgeführten anatomischen Lungenresektionen aber ausreichend wissenschaftliche Evidenz und unter aktiven Thoraxchirurgen eine hohe Akzeptanz zur Weiterentwicklung der Versorgung von Lungenkrebspatienten in Deutschland.

## Fazit

1. Die große Mehrheit (83,3 %) der befragten Thoraxchirurgen befürwortet die Einführung von Mindestmengen (MM) in der chirurgischen Versorgung des Lungenkarzinoms.
2. Die durchschnittliche Empfehlung unter den Befürwortern von MM betrug 67 anatomische Lungenresektionen bei der Diagnose Lungenkarzinom pro Jahr und Zentrum.
3. Es besteht eine hohe Korrelation zwischen der Anzahl an chirurgischen Primärfällen am jeweiligen Zentrum der Teilnehmer und der Höhe der empfohlenen MM.
4. Die aktuell gültigen Ausnahmeregelungen wurden überwiegend positiv bewertet, sodass diese auch bei der chirurgischen Versorgung des Lungenkarzinoms greifen sollten.
